# Supplementary material for: Mutations in PCYT2 disrupt etherlipid biosynthesis and cause a complex hereditary spastic paraplegia
Source: Brain. 2019 Oct 22;142(11):3382–97. doi: 10.1093/brain/awz291 (PMC6821184; doi:10.1093/brain/awz291)
Supplement: awz291_Supplementary_Data [file awz291_supplementary_data.zip › awz291-Suppl_data/Supplementary_Data4.zip!/Supplementary information 4 - plasma lipidomics.zip!/Plasma lipidomics/Control vs Patient3a/Statistical plots and data/SPH species/Boxplots.pdf]

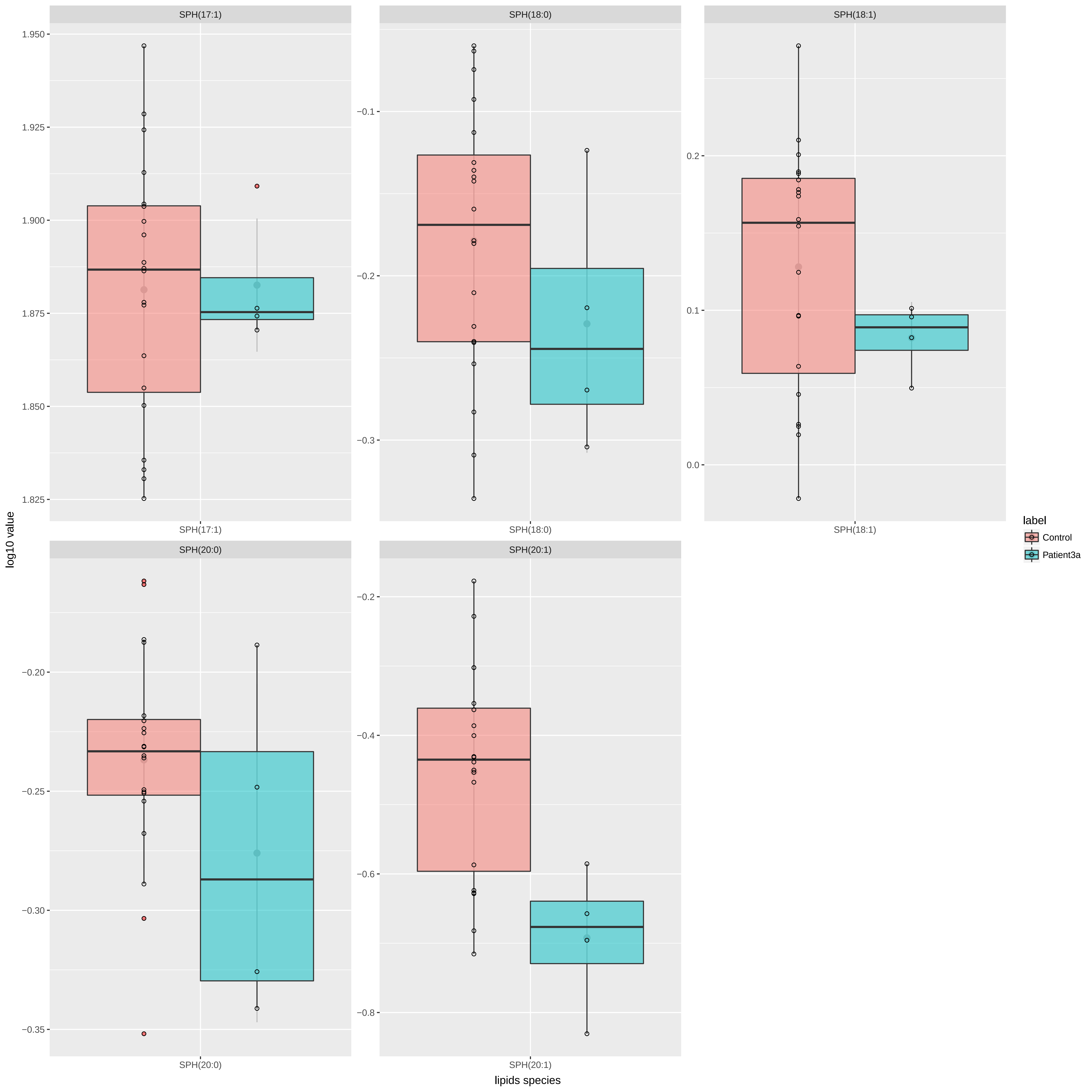

SPH(17:1)
SPH(18:0)
SPH(18:1)
SPH(20:0)
SPH(20:1)
1.950
1.925
1.900
1.875
1.850
1.825
-0.1
-0.2
-0.3
0.2
0.1
0.0
-0.20
-0.25
-0.30
-0.35
-0.2
-0.4
-0.6
-0.8
log10 value
lipids species
label
Control
Patient3a
